# The Efficacy of a Trivalent Inactivated *Salmonella* Vaccine Combined with the Live *S.* Gallinarum 9R Vaccine in Young Layers after Experimental Infections with *S.* Enteritidis, *S.* Typhimurium, and *S.* Infantis

**DOI:** 10.3390/vaccines10071113

**Published:** 2022-07-12

**Authors:** Yosef Daniel Huberman, Melanie Caballero-García, Rober Rojas, Silvia Ascanio, Leandro Hipólito Olmos, Rosana Malena, Jorgelina Lomónaco, Paula Nievas, Paula Chero, Julio Lévano-Gracía, Alfredo Mendoza-Espinoza

**Affiliations:** 1 Bacteriology, INTA EEA Balcarce, RN 226 Km 73, Balcarce CP 07620, Buenos Aires, Argentina; olmos.leandro@inta.gob.ar (L.H.O.); malena.rosana@inta.gob.ar (R.M.); lomonaco.jorgelina@inta.gob.ar (J.L.); nievas.paula@inta.gob.ar (P.N.); 2 Research and Development, Quimtia S.A, Centro Ind. Las Praderas de Lurín Mza. B. Lote 1. Lima 16, Lurín CP 15823, Peru; melanie.caballero@quimtia.com (M.C.-G.); rey.jas57@gmail.com (R.R.); silvia.ascanio@quimtia.com (S.A.); paula.chero.ancajima@gmail.com (P.C.); jlevanogarcia@gmail.com (J.L.-G.); alfredo.mendoza@quimtia.com (A.M.-E.)

**Keywords:** *Salmonella* Enteritidis, *Salmonella* Typhimurium, *Salmonella* Infantis, inactivated vaccine, chickens

## Abstract

Worldwide, poultry infections by *Salmonella* are the cause of significant economic losses, not only due to reduced production (due to fowl typhoid disease), but also considering the efforts and control measures that must be constantly applied, especially due to zoonotic serovars. Poultry is a common reservoir of *Salmonella* and its transmission into the food chain is a risk for humans. The vaccination of layers plays an important role in the overall efforts to prevent *Salmonella* infections. An inactivated trivalent vaccine was prepared with *S.* Enteritidis, *S.* Typhimurium, and *S.* Infantis strains. Infection trials were performed to evaluate the efficacy of three vaccination schedules using inactivated and live *S.* Gallinarum 9R vaccines. For this purpose, at week 5 of life, one subcutaneous dose of live *S.* Gallinarum 9R vaccine (1–5 × 10^7^ CFU) was given to Groups 1 and 2. At weeks 8 and 11 of life, chickens were also vaccinated with one (Group 1) or two (Groups 2 and 3) intramuscular doses of the inactivated oil-adjuvant trivalent vaccine (1 × 10^8^ CFU/dose of each antigen). Group 4 consisted of chickens that remained unvaccinated (control). At week 14 of life, the efficacy of the vaccination plans was evaluated in three separate inoculation trials with *S.* Enteritidis, *S.* Typhimurium, or *S.* Infantis. After vaccination with the inactivated vaccine, homologous antibody production was observed, and after challenge, a significant reduction in the faecal shedding, invasion, and colonization of *S.* Typhimurium and *S.* Infantis was achieved by all vaccination schedules, while the vaccination with at least one dose of the live *S.* Gallinarum 9R vaccine was necessary to obtain such a significant protection against *S.* Enteritidis infection.

## 1. Introduction

*Salmonella* is a Gram-negative microorganism, widely dispersed in nature and often found in the intestinal tract of animals and humans [1]. There are more than 2600 serovars that differ in their antigenic formula, according to the traditional Kauffmann–White schema for the antigenic classification of salmonellae [2]. Worldwide, poultry infections by *Salmonella* are the cause of significant economic losses, not only due to reduced production, but also considering the efforts and control measures that must be constantly applied for human health [3]. Poultry is a common reservoir of *Salmonella* and its transmission into the food chain is a risk for public health [4]. Consequently, international markets have high sanitarian demands and many restrictions that apply to food safety and public health [5]. In the United States and Europe, despite these ongoing public health and regulatory efforts to prevent and control Salmonellosis, its prevalence has remained substantial. In EU countries, Salmonellosis is the second most commonly reported foodborne gastrointestinal infection in humans [6]. In the USA, non-typhoidal *Salmonella* spp. is responsible for 11% of all foodborne illnesses and has caused 35% of hospitalizations and 28% of total deaths [7]. *S.* Enteritidis is most frequently associated with egg-related outbreaks of salmonellosis, and *S.* Typhimurium is considered the principal causative agent of foodborne salmonellosis [8]. In Europe, these two *Salmonella* serovars, Enteritidis and Typhimurium, are specifically regulated in laying flocks in all member states [9]. In Argentina, *S.* Enteritidis and *S.* Typhimurium represented more than 50% of foods contaminants from 2000 to 2005, and since 2006, *S.* Typhimurium has been the most frequent serotype isolated from humans, animals, and foods [10]. Nevertheless, the exact cause of the predominance of these *Salmonella* serovars is not yet clearly understood [11]. However, in Australia, *S.* Infantis was the most prevalent among egg-laying flocks [12] as well as in Peru, where *S.* Infantis was by far the most frequently isolated serovar and a genetic association between *S.* Infantis strains from poultry meat and human clinical isolations was observed [13,14].

Preventing flock infection with *Salmonella* is a major interest not only for poultry but also to avoid the contamination of poultry products [15]. Once *Salmonella* is introduced into a flock of laying hens, further transmission occurs via contact with infected individuals and ingestion of faecal-contaminated materials, feed, and water [16,17,18]. The potential for contact transmission of *Salmonella* may be greater when birds are subjected to stress, especially during induced moulting [16,19].

By applying strict biosecurity measures, it might be possible to achieve *Salmonella*-free farms. These measures should include tight control of feed quality and very strict hygiene, as well as investments in the appropriate installations and equipment to avoid the infiltration of rodents, insects, and wild birds into the farm. Furthermore, multi-age rearing is not recommended as new birds are rapidly becoming infected by older ones. Nevertheless, this practice is still common in many developing countries [8,20]. Consequently, due to the high costs of the implementation of efficiency measures, it is unlikely to achieve these goals [21]. On the other hand, the widespread usage of antibiotics has led to the emergence of multiple antibiotic-resistant bacteria [22,23] and many restrictions on their use [24].

Vaccination plays an important role in biosecurity systems on chicken farms to prevent *Salmonella* infections [25], and it should increase the resistance of birds to infection, thus reducing horizontal transmission, faecal excretion, and the frequency of egg contamination [26]. Killed and live attenuated vaccines have been used for controlling *Salmonella* in poultry production with extensively proven efficacy [15,26,27]. Largely, these vaccines are commercialized, but in some South American countries, availability may vary due to local sanitarian registry processes [28,29]. Killed *Salmonella* vaccines greatly help to reduce *S.* Enteritidis prevalence when implemented in laying hens flocks. These vaccines are associated with a reduction in *Salmonella* load in faeces, internal tissues, and eggs as well as lower mortality, lesions, and clinical signs in different experimental models [30]. Despite the inability of inactivated vaccines to effectively elicit a protective cell-mediated immune response, and as some bacterial antigens might be lost during the inactivation, they are regarded as considerably safe and do not present any risk of introducing live vaccine strains into the food chain [26,28,31,32]. On the other hand, live vaccines stimulate cell-mediated and humoral immune responses as they often express a wider range of antigens [28,33]. These attenuated *Salmonella* strains contain mutations or deletions in essential genes that should result in a reduced virulence but enough to induce a protective immune response [28,31]. Certain live *Salmonella* vaccines can induce cross-protection between different serotypes such as the *S.* Gallinarum 9R strain [34], which can largely protect poultry against *S.* Gallinarum infections and offer protection against infections with *S.* Enteritidis [15,35]. Inactivated vaccines were efficient in decreasing *S.* Enteritidis in broiler breeders [36], and when following the combined application of both live and killed vaccines, the protection against infection exceeded the performance of either product administered separately [37,38,39]. Most of the commercial inactivated vaccines are composed of killed cells of *S.* Enteritidis and *S.* Typhimurium [31]; others include *S.* Infantis [39], *S.* Heidelberg, or even local or regional strains of a certain serovar of importance [40].

The present trials aimed to evaluate the efficacy of a trivalent inactivated vaccine prepared with antigens of *S.* Enteritidis, *S.* Typhimurium, or *S.* Infantis with or without an initial vaccination dose of live *S.* Gallinarum 9R vaccine after experimental inoculation of young layers with either *S.* Enteritidis, *S.* Typhimurium, or *S.* Infantis

## 2. Materials and Methods

### 2.1. Salmonella Strains

Three *Salmonella* strains from Peru were used; *S.* Enteritidis strain Q391 and *S.* Typhimurium strain Q782 were isolated from layers, while *S.* Infantis strain Q360 was isolated from broilers. These strains were used for the preparation of the Bacterin and for the experimental inoculations.

### 2.2. Chickens

*Salmonella*-free birds were used in the three trials (Table 1). One-day-old female chicks were housed immediately after hatching: in Trial 1, Hy-line chickens (Produss, Lima, Peru) while in Trials 2 and 3, Lohmann Brown chickens were used (La Camila, Entre Ríos, Argentina). Before entering the rearing facilities, every bird was wing-tagged and *Salmonella*-free status was confirmed by bacteriologically culturing of meconium samples from all birds. Afterwards, weekly cloacal swabs were randomly taken from ten chickens per group. These meconium samples and swabs were pooled into 40 mL of Tetrathionate broth supplemented with Brilliant Green (TB) (Oxoid, CM0029) and, after overnight incubation was sub-cultured onto XLD (Difco, 278850) + 0.46% Tergitol 4 (Sigma, 100H0494) (XLDT4) agar.

### 2.3. Feed and Water

Animals received ad libitum *Salmonella*-free balanced food based on a vegetable protein diet, free from meat or fishmeal, without adding any antibiotics or coccidiostats. To ensure the absence of *Salmonella* spp. in feed, cultures of all batches using lactose broth were performed as previously described [41,42].

### 2.4. Vaccines and Vaccination

An oil-adjuvant (W/O/W), trivalent, inactivated vaccine was prepared using the three aforementioned *Salmonella* strains. Each strain was grown separately, inactivated with formalin 0.1% (*v*/*v*), and added to the vaccine at a final concentration of 1 × 10^8^ colony-forming units (CFU)/dose. On the other hand, the commercial vaccine based on *S.* Gallinarum strain 9R (1–5 × 10^7^ CFU/dose) also contained inactivated antigens of *Escherichia coli* (Inmuno Tifo C^®^, QUIMTIA SA, Lima, Peru).

Upon arrival, chicks were randomly distributed into four experimental groups as shown in Table 1. Birds of Groups 1 and 2 received one subcutaneous dose of the 9R vaccine at week 5 of life. Afterwards, at week 8 of life, the pullets of Groups 2 and 3 were intramuscularly vaccinated with the inactivated trivalent vaccine. Finally, at week 11 of life, all hens of Groups 1, 2, and 3 were vaccinated intramuscularly with the inactivated trivalent vaccine. The birds of Group 4 remained unvaccinated and were used as the negative control.

### 2.5. Preparation of the Inoculum and Avianization

Each *Salmonella* strain was thawed from liquid nitrogen and cultured onto an XLDT4 agar plate. After overnight incubation at 37 °C, one colony was used to seed a Brain Heart Infusion broth (BHI) (Oxoid, CM1135), which was incubated overnight at 37 °C. Before the beginning of the trials, the virulence of the three *Salmonella* strains was enhanced as previously described [43,44]. Briefly, each strain was inoculated by gavage into the crop using two *Salmonella*-free one-day-old chicks. After two days, these chicks were euthanized, and their livers and spleens were cultured onto XLDT4 plates that were processed as before. For standardization of the proceedings, the growth from the agar plates was collected, aliquoted, and kept frozen in liquid nitrogen until used.

### 2.6. Bacteriology

In all trials, individual cloacal swabs were cultured in 5 mL TB that was incubated for 48 h at 37 °C. After euthanasia, livers and spleens were macerated and diluted 1:1 (*w*/*v*) using sterile BHI, while caecum contents samples were diluted 1:2 (*w*/*v*) using TB in sterile plastic bags. After homogenizations, five log10 dilutions were prepared for each sample using BHI (for livers and spleens) or TB (for caecum content), which were incubated overnight for 48 h, respectively, at 37 °C. After incubation, all tubes were vortexed and subcultured onto XLDT4 agar plates as before. The presence of the challenge strain was registered for each tube.

### 2.7. Pre Trials

To establish the minimum infective dose 100% of each strain, a pre-trial was conducted. For each *Salmonella* strain, an inoculum was prepared as above, and two log10 dilutions (1:10 and 1:100) were prepared in sterile BHI. These dilutions were used to inoculate fifteen 12-week-old birds that were divided into three groups of five birds each. These birds were orally inoculated by gavage into the crop with 0.5 mL of one of the correspondent dilutions. On day 3 (Trials 2 and 3) or days 3 and 5 (Trial 1) post-inoculation, all birds were sampled by cloacal swabbing and euthanized. Livers and spleens were cultured as before. The results from the pre-trials are shown in Table S1.

### 2.8. Experimental Infections with Salmonella

Three infection trials were conducted. At week 14 of life, the hens were orally inoculated with the correspondent *Salmonella* strain with 0.5 mL of the inoculum by gavage into the crop (Table 1).

### 2.9. Cloacal Swabbing and Samplings

Individual cloacal swabs were taken from all birds on days 3, 6, 9, 12, and 15 post-challenge. In Trial 1, cloacal swabs were also taken on days 2, 4, and 7 post-challenge. Each swab was deposited into a tube containing 3 mL TB that was cultured as before. On days 3, 6, 9, 12, and 15 post-challenge, five (Trial 1) or six (Trials 2 and 3) birds from each group were sacrificed by decapitation. Livers, spleens, and caecum contents were cultured as above (without dilutions). Positive/negative results were recorded.

### 2.10. Serology

Blood samples were taken before the first vaccination at week 5 of life and approximately every 10 days until week 14 of life when birds were challenged. Thereafter, in Trial 1, blood samples were taken on day 15 post-challenge, while in Trials 2 and 3, blood samples were taken on days 3, 6, 9, 12, and 15 post-challenge. Blood was taken from the wing veins of the chickens using 2.5 mL syringes and 30G needles [45] and transferred into 5 mL plastic tubes. Afterwards, sera were separated and kept frozen at −20 °C in 1.5 mL tubes until usage. Tittering of anti-*Salmonella* antibodies was evaluated using ELISA kits Salm Gp D (https://www.biochek.com/poultry-elisa/salmonella-group-d-antibody-test-kit/ accessed on 30 June 2022) and Salm Gp B (https://www.biochek.com/poultry-elisa/salmonella-group-b-antibody-test-kit/, accessed on 30 June 2022, Biochek, Scarborough, ME, USA) specific for *S.* Enteritidis (Trial 1) and *S.* Typhimurium (Trial 2), respectively. On the other hand, in Trial 3, the anti-*Salmonella* antibodies titres in sera were evaluated by the micro-agglutination test (MAT) [46,47,48]. For that purpose, the *S.* Infantis was cultured in BHI broth, inactivated, centrifuged, resuspended in PBS + Thimerosal (0.01%), and adjusted to a final concentration of 1 × 10^9^ bacteria/mL (0.5 at OD600m). Afterwards, sera samples were diluted at 1:10 and ten Log2 dilutions were prepared and incubated (overnight at 37 °C) with the inactivated antigen. Titres were calculated as the highest log2 dilution with a positive reaction.

### 2.11. Statistics

The chi-square test of independence was performed to compare de re-isolation rates of *Salmonella* sp. among the four experimental groups in each trial (significance level α = 0.05).

### 2.12. Animal Welfare

Handling of birds was performed according to the Guide for the Care and Use of Laboratory Animals [49], and euthanasia was performed according to the American Veterinary Medical Association’s Manual for euthanasia [50]. The performance of these trials was previously evaluated and approved by the Regional Council of the National Institute for Agro-Technology (CICUAE INTA CeRBAS), and the trials were performed accordingly (approvals numbers 01815 and 05016).

## 3. Results

### 3.1. Cloacal Swabs

The recovery rates of the challenge strains in the three trials are shown in Table 2. In Trial 1, significantly more cloacal swabs from the unvaccinated birds (Group 4) were positive for the challenge strain (140 positive swabs out of 191) in comparison with the other groups, with 124, 63, and 107 positive swabs from Groups 1, 2, and 3, respectively, which were also statistically different. Similarly, in trials 2 and 3, significantly more cloacal swabs from the unvaccinated birds (Group 4) were positive for the challenge strain (17/90 and 23/90, respectively) in comparison with the other groups in the same trial. In trial 2, only one positive swab (out of 81 samples) was obtained from birds of group 3, significantly lower than the ones from groups 1 and 2 (7/90 each). Furthermore, in trial 3, no statistical differences were found among groups 1, 2, and 3 (5/90, 3/90, and 5/90, respectively). Information about the daily results of samplings is available in Supplementary Table S2.

### 3.2. Salmonella Recovery from Livers, Spleens, and Caecum Contents

The recovery rates from livers, spleens, and caecum content and the number of positive birds are presented in Table 3, while more information is available in Supplementary Table S3.

**Trial 1.** Significantly fewer positive livers and spleens from Groups 1 and 2 were found in comparison with those from Groups 3 and 4, which did not statistically differ among themselves. Similarly, fewer caecum contents from Group 2 were positive in comparison with those from Groups 3 and 4, which did not statistically differ among themselves. The recovery rate of *S.* Enteritidis from caecum contents in Group 1 did not differ statistically from the other three experimental groups. Overall, significantly fewer positive samples (30/75) were obtained from birds of Group 2 in comparison with the other three groups, followed by Group 1 (41/75), which was significantly lower in comparison with Groups 3 and 4 (62/75 and 64/75, respectively), which did not statistically differ among themselves. Furthermore, all of the chickens from Group 4 were positive, significantly more so than Groups 1 and 2. In Group 3, the number of positive birds did not statistically differ from the other groups.

**Trial 2.** Significantly more livers, spleen and caecum contents from Group 4 were positive in comparison with the other three vaccinated groups, which did not statistically differ among themselves, except for Group 3, which had significantly more positive spleens in comparison with Groups 1 and 2. The total recovery rate of *Salmonella* from all samples from birds of Group 1 (7/90) and Group 2 (6/90) did not statistically differ among themselves but were significantly lower in comparison with Group 3 (17/84), which was also significantly lower than the total recovery of *S.* Typhimurium from birds of Group 4 (43/90). All vaccinated groups presented significantly fewer positive birds in comparison with the unvaccinated Group 4.

**Trial 3.** Significantly more livers, spleen and caecum contents from Group 4 were positive in comparison with the other three vaccinated groups, which did not statistically differ among themselves, except for the livers from Group 3 that did not differ from the other groups. Overall, significantly more positive samples from the unvaccinated Group 4 (62/90) were recovered in comparison with the other three groups. In addition, there were more positive birds in the unvaccinated group in comparison with all of the vaccinated groups, while in Group 1, the number of positive birds was significantly higher than in Groups 2 and 3.

### 3.3. Serology

**Figure 1:**
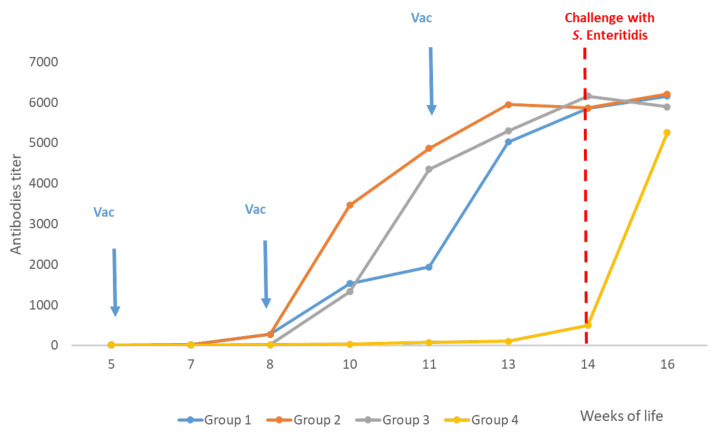
Trial 1—Antibodies titres. Measurements of antibody titres in vaccinated and non-vaccinated chickens. Challenge with *S.* Enteritidis (SE) was carried out in week 14 of life (red line). Blood samples were taken approximately every 10 days starting at week 5 until week 16 of life (two weeks post-challenge) when all birds were euthanized. Sera were analysed by ELISA (Salm Gp D, Biochek).

**Figure 2:**
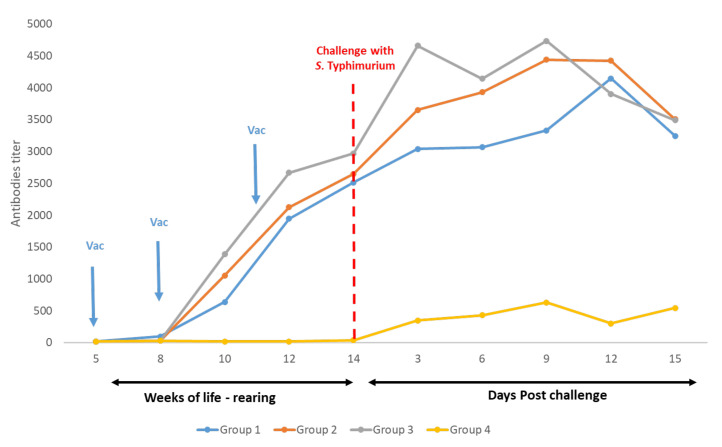
Trial 2—Antibodies titres. Measurements of antibody titres in vaccinated and non-vaccinated chickens. Challenge with *S.* Typhimurium (ST) was carried out in week 14 of life (red line). Blood samples were taken approximately every 10 days starting at week 5 of life, and after the challenge, samples were taken every three days until day 15 post-challenge, when all birds were euthanized. Sera were analysed by ELISA (Salm Gp B, Biochek).

**Figure 3:**
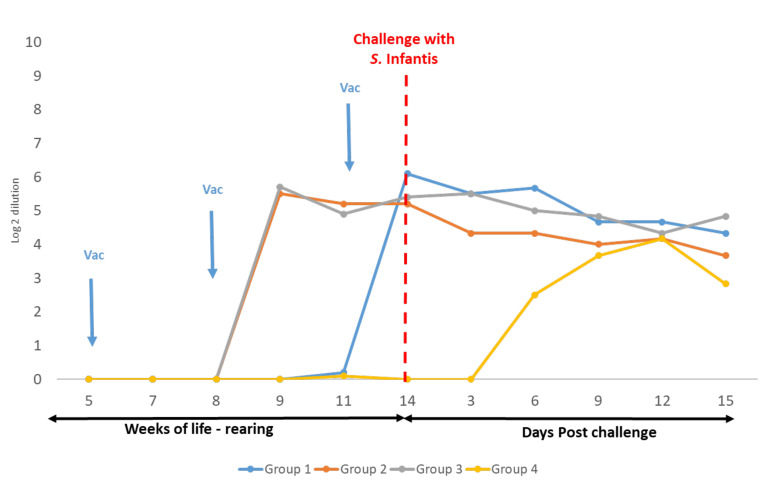
Trial 3—Micro-agglutination test (MAT). Measurements of titres in vaccinated (Groups 1, 2, and 3) and non-vaccinated (Group 4) chickens. Blood samples were taken approximately every 10 days starting at week 5 of life. Ten Log2 dilutions of each serum were incubated with antigens of *S.* Infantis. Antibody titres were calculated as the highest log2 dilution that reacted.

The ELISA results for Trials 1 and 2 are presented in Figure 1 and Figure 2, respectively, and MAT results for Trial 3 are available in Figure 3.

In Trials 1 and 2, the vaccination at week 5 of life with the live vaccine of *Salmonella* Gallinarum 9R caused a very low production of antibodies against *S.* Enteritidis or *S.* Typhimurium, respectively, which was evidenced only from week 10 of life (Group 1). Nevertheless, in Trial 3, a similar presence of antibodies against *S.* Infantis was not observed by MAT in this group.

On the other hand, in all trials, 10 days after the first vaccination with the inactivated vaccine at week 8 of life, antibody titres were detected in Groups 2 and 3. Similarly, in all trials, after the second vaccination with the inactivated vaccine at week 11 of life, homologous antibodies titres were also detected in Group 1. In Trials 1 and 3, on day 15 post-challenge, antibodies titres against *S.* Enteritidis or *S.* Infantis, respectively, from the unvaccinated chickens (Group 4) were similar to the three vaccinated groups. On the contrary, in Trial 2, antibodies against *S.* Typhimurium were very low in blood samples from the unvaccinated chickens taken after the challenge.

## 4. Discussion

*Salmonella* control in poultry farms should be based on the application of strict biosecurity measures. These include major efforts in controlling housing conditions, feed quality, strict hygiene, plagues control, contact with wildlife animals, and, where possible, avoiding multi-age rearing [8,20]. In many countries, these measures are too expansive and are not being implemented [21]. Vaccination against *Salmonella*-nonspecific host serovars was reported with variable success rates. Inactivated vaccines produce good immune responses but generally lack cross-protection against other serovars [37]. While vaccination with homologous serovars can show cross-protection within the same serologic group [51], multivalent inactivated vaccines, prepared from a mixture of strains from different serovars, provide an expanded spectrum of protection [39,52].

In South American countries, layers are commonly vaccinated with the 9R vaccines against fowl typhoid caused by *S.* Gallinarum [10]. Furthermore, cross-protection by the 9R vaccine against *S.* Enteritidis was reported as both serovars belong to the same serogroup [53]. The vaccination schedules that were tested in the present study consisted of vaccination with the live *S.* Gallinarum 9R in week 5 of life in combination with one or two doses of the inactivated trivalent vaccine in weeks 8 and 11 of life (Groups 1 and 2, respectively). Usually, vaccination with the 9R vaccine is administered at 8 weeks of age, but this vaccine could be administered as early as 4 weeks of life [54,55]. In the present study, vaccination with the 9R vaccine was administered at week 5 of life and no negative effects were observed, nor was the excretion of the vaccine strains detected in faecal samples.

Killed vaccines were associated with decreased incidences of *S.* Enteritidis infection in Dutch broiler breeder flocks and *S.* Enteritidis contamination in eggs from Japanese laying flocks [35,56]. In Trial 1, to obtain a significant reduction in the invasion and colonization of *S.* Enteritidis, the use of the live *S.* Gallinarum 9R vaccine with the addition of at least one dose of the inactivated trivalent vaccine was required. Nevertheless, there was a significant reduction in the number of samples positive for *S.* Typhimurium and *S.* Infantis (Trials 2 and 3, respectively) in comparison with the unvaccinated birds. This reduction was also observed in the number of positive birds (that had at least one positive sample after euthanasia). Furthermore, the protection afforded by two doses of the inactivated vaccine (Group 3) was good enough even without the previous vaccination with the live *S.* Gallinarum 9R vaccine (Groups 1 and 2). These results should not be surprising, as the use of the 9R vaccine did not protect against intestinal colonization by *S.* Typhimurium or *S.* Infantis [57]. In a similar trial, performed by Deguchi et al. (2009), using an inactivated vaccine with the same three *Salmonella* serovars, there was also a reduction in the number of *S.* Enteritidis, *S.* Typhimurium, *S.* Infantis, and *S.* Heidelberg from faeces, caecum colonization, and organ invasion. Nevertheless, the *S.* Enteritidis that was used was isolated from humans [39].

Environmental contamination, including floors, feeders, and drinkers is a result of the faecal shedding of *Salmonella* by infected hens [58]. In the present trials, the three vaccination schedules were helpful in significantly reducing the excretion of *Salmonella*, and thus contributing to the reduction in the dissemination of *Salmonella* on farms. El-Enbaawy et al. [47] used bivalent (*S.* Enteritidis and *S.* Typhimurium) and polyvalent (*S.* Enteritidis, *S.* Typhimurium, *S.* Infantis, and *S.* Meleagridis) inactivated vaccines and obtained a significant reduction in *Salmonella* excretion after a simultaneous challenge with these four serovars.

In the present trials, antibody production was evidenced after vaccination with the inactivated vaccines during rearing. These were measured by ELISA in Trials 1 and 2, showing the efficiency of the inactivated vaccine to produce antibodies against *S.* Enteritidis y *S.* Typhimurium, respectively. As no commercial ELISA was available for the detection of antibodies against *S.* Infantis, the blood samples from Trial 3 were processed by the MAT technique. This technique is not as accurate as ELISA, but it provides a good qualitative tool to compare the levels of antibody production among different vaccination schedules. Furthermore, it has some advantages of savings in time, space, and cost [46]. It was revealed that the inactivated vaccine enabled the production of antibodies against *S.* Infantis with similar levels that were detected by ELISA against *S.* Enteritidis and *S.* Typhimurium. After the challenge, in the three trials, homologous antibodies were produced in the unvaccinated birds. In Trials 1 and 3, the antibody titres of unvaccinated chickens against *S.* Enteritidis and *S.* Infantis, respectively, reached similar levels of antibodies in comparison with the vaccinated chickens. Hence, it was shown that the three vaccination schedules were efficient for producing enough antibodies, similar to the levels of antibodies that may be produced by the chickens when a pathogenic strain is introduced. Differently, after challenge with *S.* Typhimurium, the titres of antibodies in unvaccinated chickens were lower in comparison with the other vaccinated chickens (Trial 2). This is probably because more time was needed to reach the same levels of antibodies.

Under the conditions of this study, the results suggest that the inactivated trivalent *Salmonella* vaccine can be an effective tool for controlling *S.* Typhimurium and *S.* Infantis. It has also contributed to significantly reducing the excretion of *S.* Enteritidis. Nevertheless, the combination of the live 9R vaccine with at least one dose of the inactivated triple *Salmonella* vaccine filled this gap of protection against *S.* Enteritidis, achieving a significant reduction in faecal shedding. Furthermore, the use of one dose of the 9R vaccine with only one dose of the inactivated vaccines might be effective, and thus help to reduce the costs of vaccinations, especially in countries where high costs of vaccination might be a limiting factor.

Future trials should include challenges with pathogenic *S.* Gallinarum. It might also be interesting to compare the inactivated vaccine, which was prepared with local Peruvian strains, with other available inactivated multivalent vaccines that are prepared with international strains. Field trials in poultry farms with a history of *Salmonella* can also be included and may shed more light on the efficacy and duration of protection as well as antibodies production during the egg production period.

## Figures and Tables

**Table 1:** Vaccination and challenge. Schedule and number of birds. The chickens were vaccinated with the 9R vaccine and with the trivalent inactivated vaccine. At week 14 of life, in each trial, the chickens were inoculated by gavage into the crop with 0.5 mL of the correspondent strain.

Group	Age of Vaccination (Weeks)			*n*		
				Trial 1	Trial 2	Trial 3
				Challenge Strain		
	5	8	11	*S.* Enteritidis	*S.* Typhimurium	*S.* Infantis
1	9R	-	Inactivated	32	30	30
2	9R	Inactivated	Inactivated	32	30	30
3	-	Inactivated	Inactivated	32	28	30
4	-	-	-	32	30	30
	Inoculation Dose (CFU) *			1 × 10^7^	4 × 10^7^	6 × 10^7^

* Colony-forming units.

**Table 2:** Cloacal swabs. Cloacal swabs were taken from all birds on days 3, 6, 9, 12, and 15 post-challenge. In Trial 1, cloacal swabs were also taken on days 2, 4, and 7 post-challenge.

Group	Number of Positive Cloacal Swabs Samples		
	Trial 1	Trial 2	Trial 3
	*S.* Enteritidis	*S.* Typhimurium	*S.* Infantis
1	124/191 ^a^	7/90 ^a^	5/90 ^a^
2	63/191 ^b^	7/90 ^a^	3/90 ^a^
3	106/191 ^c^	1/81 ^b^	5/90 ^a^
4	140/191 ^d^	17/90 ^c^	23/90 ^b^

^a,b,c,d^ Isolation rates in the same column (trial) without common superscripts differ statistically using the chi^2^ test (*p* < 0.05).

**Table 3:** The total isolation rates of *S.* Enteritidis, *S.* Typhimurium, or *S.* Infantis from livers, spleens, and caecum contents after challenge. Samples were taken from five (Trial 1) or six (Trials 2 and 3) birds of each group on days 3, 6, 9, 12, and 15 post-challenge. A chicken was considered positive if at least one sample was positive.

Number of Trial/*Salmonella* Strain	Group	*n*	Number of Positive Samples				Number of Positive Birds
			Liver	Spleen	Caecum Contents	Total	
Trial 1 *Salmonella* Enteritidis	1	25	8 ^a^	13 ^a^	20 ^ab^	41 ^a^	21 ^a^
	2	25	7 ^a^	8 ^a^	15 ^a^	30 ^b^	18 ^a^
	3	25	18 ^b^	22 ^b^	22 ^b^	62 ^c^	23 ^ab^
	4	25	17 ^b^	24 ^b^	23 ^b^	64 ^c^	25 ^b^
Trial 2 *Salmonella* Typhimurium	1	30	1 ^a^	1 ^a^	5 ^a^	7 ^a^	6 ^a^
	2	30	2 ^a^	0 ^a^	4 ^a^	6 ^a^	6 ^a^
	3	28	4 ^a^	7 ^b^	6 ^a^	17 ^b^	11 ^a^
	4	30	10 ^b^	20 ^c^	13 ^b^	43 ^c^	23 ^b^
Trial 3 *Salmonella* Infantis	1	30	3 ^a^	10 ^a^	13 ^a^	26 ^a^	18 ^a^
	2	30	1 ^a^	8 ^a^	7 ^a^	16 ^a^	13 ^b^
	3	30	6 ^ab^	7 ^a^	6 ^a^	19 ^a^	13 ^b^
	4	30	13 ^b^	26 ^b^	23 ^b^	62 ^b^	29 ^c^

^a,b,c^ Isolation rates and the number of positive birds in the same column (for each trial) without common superscripts differ statistically using the Chi^2^ test (*p* < 0.05).

## Data Availability

Not applicable.

## Supplementary Materials

The following supporting information can be downloaded at: https://www.mdpi.com/article/10.3390/vaccines10071113/s1, Table S1: Pre-trials to establish the minimum infective dose 100%, Table S2: Cloacal swabs, Table S3: The number of positive samples of livers, spleens, and caecum contents after challenges at week 14 of life in three experimental infection trials.
