# Supplementary material for: The Efficacy of a Trivalent Inactivated Salmonella Vaccine Combined with the Live S. Gallinarum 9R Vaccine in Young Layers after Experimental Infections with S. Enteritidis, S. Typhimurium, and S. Infantis
Source: Vaccines (Basel). 2022 Jul 12;10(7):1113. doi: 10.3390/vaccines10071113 (PMC9323758; doi:10.3390/vaccines10071113)
Supplement: Supplementary file 1 [file vaccines-10-01113-s001.zip › vaccines-1777321-supplementary.pdf]

Table S1. Pre-trials to establish the minimum infective dose 100%. Twelve-week-old birds were orally inoculated by gavage into the crop with 0.5 mL of one of the correspondent inocula. Birds were sampled by cloacal swabbing and euthanized. Livers, spleens, and caecum content were cultured. For each sample, five log10 dilutions were prepared.

Number of Trial / *Salmonella* Strain	Inoculation Dose	Bird Wing-Tag Number	Number of Positive Tubes			Cloacal Swabs (Day Post-Inoculation)	
			Liver	Spleen	Caecum Contents	Day 3	Day 5
Trial 1 *Salmonella* Enteritidis	1 x 10^9	20	+++	+++	++++++	+	+
		4559	++	+++	++++++	+	+
		4580	+++	+++	++++++	+	+
		8114	+++	+++	++++	+	+
		8123	+++	+++	++++++	+	+
	1 x 10^8	12	++	+++	++++++	+	+
		18	+++	+++	++++++	+	+
		4541	+++	++	+++++	+	+
		4574	+++	+++	++++	+	+
		4586	+++	+++	++	+	+
	1 x 10^7	2006	++	+++	++++++	+	+
		2015	+++	+++	++++++	+	+
		4546	+++	++	++	+	+
		6105	+++	+++	++++++	+	+
		6129	++	+++	-	+	+
Trial 2 *Salmonella* Typhimurium	1 x 10^9	4413	+	-	+++	+	ND
		4436	-	+	+++	+	ND
		4448	-	-	+++	+	ND
		4452	-	-	+++	+	ND
		4473	+	+	+++	+	ND
	1 x 10^8	4424	-	+	+	+	ND
		4469	-	-	+++	+	ND
		4472	-	-	+++	+	ND
		4492	-	-	+++	+	ND
		4499	-	-	+++	+	ND
	1 x 10^7	4495	-	-	-	+	ND
		4500	-	-	-	+	ND
		4410	-	-	-	-	ND
		4463	-	-	-	-	ND

		4445	-	-	+	+	ND
Trial 3 *Salmonella* Infantis	1×10^{9}	11	++++	++++++	+++	-	ND
		12	++++++	++++++	++++	+	ND
		13	+++++	++++++	++++	-	ND
		14	++++++	++++++	-	+	ND
		15	+	++++++	++++	-	ND
	1×10^{8}	6	+	++++++	+	-	ND
		7	+	++++++	++	+	ND
		8	+++	++++	++++++	+	ND
		9	+++++	++++++	-	-	ND
		10	++	++++++	-	-	ND
	1×10^{7}	1	+	+++++	+++	-	ND
		2	+	++++++	+	-	ND
		3	+	+++	-	-	ND
		4	++	++	++	-	ND
		5	-	+	-	-	ND

ND – Not done. + Number of positive log10 tubes that were *Salmonella*-positive.

Table S2. Cloacal swabs. Vaccinated and unvaccinated chickens were inoculated at week 14 of life and cloacal swabs were taken from all birds on days 3, 6, 9, 12, and 15 post-challenge. In Trial 1, cloacal swabs were also taken on days 2, 4, and 7 post-challenge. Positive/negative results were recorded.

**Number of Trial / *Salmonella* Strain**	***Group***	**Positive Cloacal Swabs / Total Number of Samples**								**Total**
		**Day Post-Inoculation**								
		**2**	**3**	**4**	**6**	**7**	**9**	**12**	**15**	
Trial 1 *Salmonella* Enteritidis	1	27/32^{a}	17/32^{a}	4/27^{a}	18/27^{a}	19/22^{a}	20/22^{a}	13/17^{a}	6/12^{a}	124/191^{a}
	2	20/32^{b}	9/32^{b}	3/27^{a}	9/27^{b}	7/22^{b}	6/22^{b}	7/17^{b}	2/12^{b}	63/191^{b}
	3	27/32^{a}	16/32^{a}	6/27^{a}	17/27^{a}	13/22^{c}	14/22^{c}	10/17^{ab}	4/12^{ab}	107/191^{c}
	4	32/32^{c}	12/32^{ab}	14/27^{b}	21/27^{c}	21/22^{a}	19/22^{a}	13/17^{a}	5/12^{ab}	140/191^{d}
Trial 2 *Salmonella* Typhimurium	1	ND	4/30^{a}	ND	2/24	ND	1/18^{ab}	0/12	0/6	7/90^{a}
	2	ND	5/30^{a}	ND	2/24	ND	0/18^{a}	0/12	0/6	7/90^{a}
	3	ND	1/28^{a}	ND	0/22	ND	0/16^{a}	0/10	0/5	1/81^{b}
	4	ND	11/30^{b}	ND	1/24	ND	4/18^{b}	1/12	0/6	17/90^{c}
Trial 3 *Salmonella* Infantis	1	ND	0/30^{a}	ND	0/24^{a}	ND	1/18	4/12	0/12	5/90^{a}
	2	ND	1/30^{ab}	ND	0/24^{a}	ND	0/18	2/12	0/12	3/90^{a}
	3	ND	3/30^{ab}	ND	2/24^{a}	ND	0/18	0/12	0/12	5/90^{a}
	4	ND	7/30^{b}	ND	9/24^{b}	ND	4/18	2/12	1/12	23/90^{b}

ND – Not done. ^{a,b,c,d} Isolation rates in the same column (trial) without common superscripts statistically differ using the chi^{2} test ($P<0.05$).

Table S3. The number of positive samples of livers, spleens, and caecum contents after challenges at week 14 of life in three experimental infection trials.

Number of Trial / *Salmonella* Strain	Group	Day Post-Inoculation														
		3			6			9			12			15		
		Liver	Spleen	Caecum Contents	Liver	Spleen	Caecum Contents	Liver	Spleen	Caecum Contents	Liver	Spleen	Caecum Contents	Liver	Spleen	Caecum Contents
Trial 1 *Salmonella* Enteritidis	1	3	2	5	2	3	5	0	2	4	1	4	3	2	2	3
	2	1	0	4	2	2	3	2	3	3	0	2	4	2	1	1
	3	4	4	4	5	5	5	4	4	3	3	5	5	2	4	5
	4	5	5	5	5	5	5	5	5	4	2	5	5	0	4	4
Trial 2 *Salmonella* Typhimurium	1	0	0	1	0	1	3	0	0	0	1	0	1	0	0	0
	2	0	0	1	1	0	0	0	0	0	0	0	3	1	0	0
	3	0	0	0	2	3	3	2	2	0	0	1	3	0	1	0
	4	2	2	3	2	6	2	6	6	4	0	4	4	0	2	0
Trial 3 *Salmonella* Infantis	1	2	0	2	1	1	3	0	5	3	0	0	3	0	0	0
	2	0	1	3	1	1	3	0	5	0	0	1	1	0	0	0
	3	2	1	2	0	1	0	4	3	1	0	1	1	0	1	2
	4	4	5	6	4	4	6	3	6	4	2	5	4	0	6	3
